# Dental Societies and Dental Literature

**Published:** 1885-07

**Authors:** C. J. Ellis


### DENTAL SOCIETIES AND DENTAL LITERATURE.

Read Before the Eighth District Dental Society of the State of New York.

BY C. J. ELLIS, D. D. S.

It will be seen that this paper is entitled "Dental Societies and Dental Literature." It may be thought that this necessarily implies a division of subjects, and yet when we think the matter over they appear inseparable. The one chronicles the work of the other. And here let me make what may seem a radical statement. We could wipe out all dental colleges and text books, and get along better than if deprived of all dental societies and dental journals. How many dentists to-day consult dental text books and college note books as compared with the continual reference to the work of to-day, as mirrored in society meetings and dental journals. Dental therapeutics has no history or solid foundation, but is rapidly acquiring one, as the investigations of individual members are made public in society meetings and reports. All our present knowledge of pyorrhœa alveolaris and its treatment is gathered from the few who have made it a special study and given us the results in dental societies and journals.

When I reflect upon the great disadvantages under which dentists labored twenty-five or fifty years ago, I am thankful for the privileges we of to-day enjoy, and the benefits derived from the constant interchange of ideas. The more professional one becomes, the more ready is he to impart knowledge ; hence the multiplication of societies and journals.

One great advantage of the professional man in the city, is the ability to call upon brothers at his elbow for information. How many points in dentistry have been developed by one man only! It is a hint here and a suggestion there, added to the original idea, that makes complete success. This is constantly illustrated by the remarks of successful dentists, whom you may frequently hear say: "I obtained that idea from Dr. Blank." And yet, notwithstanding all the benefits to be derived from society meetings, it seems to be but an intelligent minority that avails itself of their privileges. We can but hope that, as dentistry grows older, a larger proportion of the young men coming into the profession will identify themselves with professional societies. Many who would otherwise attend are doubtless debarred by a sense of their inability to secure membership without a preliminary examination.

Much of the benefit that is derived from society meetings is due the journals, for they give to the profession at large the thoughts that otherwise would be received by but the few in actual attendance. Reading these reports, it is easy to note the dogmatic positivists and the speculative theorists, always ready to split a hair and then to spend a year in determining the largest half. Such men are to be found in every society.

Any society holding monthly meetings can keep a firmer grasp upon a subject than is possible when the meetings occur only once or twice a year, and when there are so many topics to discuss. I wish some plan could be devised by which we could know what direction discussions will take at the next meeting, so that we might be adding to our knowledge of those particular subjects and be enabled to discuss them understandingly.

In all society meetings there is a tendency for certain individuals to monopolize the time to the exclusion of others who might be induced to speak, and thus make the discussion more general. This may be of benefit to the talkers, but it is not for the best interests of any society. The more general discussions are, the greater the amount of good accomplished. There is something audacious in the confidence that allows one or two speakers to discuss and dismiss subject after subject, while a score of members say nothing. This may be unavoidable, owing to the modesty inherent in the dental bosom, but, I say again, it is not for the best interest of any society. A question should be of sufficient interest, if debated at all, to call out half a dozen members, and all sides should be discussed, and that very briefly.

There may be those here who, after looking at the title of my paper, infer that I shall attempt to review, in a general sense, the quality of the work we get in our publications. Such was not my intention, and yet I will own to having positive opinions of some of our writers. I never read one of Dr. Beers' graphic articles in *The Century,* which so vividly portray out-door sports in Canada, but I congratulate myself that he is one of us. And not long ago I noted a thoughtful article in the INDEPENDENT PRACTITIONER from the same pen. But I will not follow this line of thought farther now, for it would take a separate article to specify those worthy of mention as writers.

General literature to-day is dotted with contributions from the medical fraternity, and I hope I will live to see an equal number of dentists known as writers. The more widely any dentist is known for his culture, the more the profession is elevated. Dentistry of to-day is but in its infancy. I mean not only as to the improvements, but its hold upon the public. We have a long fight, gentlemen, to earn the whole confidence and esteem of the public. We cannot do it by legislation, or by the so-called "regular" system, as with the medical fraternity. Rather must it be accomplished by earnest effort in all our societies and journals, to educate ourselves aright, to stimulate each other, to cultivate wide and liberal views on all questions pertaining to our profession, and so to bring the public gladly to acknowledge the dentist as in truth a professional man.

In every town and village the dentist should deliver practical lectures before the schools, and so educate the youth to a care of the teeth. There are some communities so educated, but they are few. The public at large has little care for their teeth, and we as dentists are responsible for this condition if we do not try to educate them out of it. The colleges are doing their work, but we as individuals have a greater one. I see the evidence every day of dental ignorance, the seed of which was sown years ago by ignorant dentists. If we admit that the life and progress of dentistry is in the keeping of the societies and journals, let us as individual members at least be content with no half-way work or measures, but each strive to do his whole duty by both.

